# Panorama epidemiológico de las enfermedades transmitidas por vectores: lecciones aprendidas y retos para romper el círculo

**DOI:** 10.7705/biomedica.7331

**Published:** 2023-12-01

**Authors:** Julio César Padilla-Rodríguez

**Affiliations:** 1 Consultor, Red Nacional de Innovación e Investigación en Malaria

Actualmente, en los perfiles de salud-enfermedad de la población en la mayoría de los países de la región de las Américas, se observa la coexistencia y la simultaneidad de enfermedades transmisibles endemo-epidémicas persistentes, la reemergencia de enfermedades que se habían controlado y la aparición cada vez más frecuente de otras nuevas, con eventos crónicos no transmisibles prevalentes, las cuales imponen una importante carga social y económica en la población expuesta y en las instituciones responsables. Entre las enfermedades transmisibles, se destacan las transmitidas por vectores que mantienen una alta prevalencia. Según la OPS/OMS, las enfermedades transmisibles en las Américas ponen en riesgo la salud de una de cada dos personas y una gran proporción de estas son transmitidas por artrópodos de importancia en salud pública, como las enfermedades transmitidas por vectores o metaxénicas ^1^^,^^2^.

De todas estas, las más frecuentes son la malaria y el dengue. En zonas rurales tropicales de la región, las enfermedades parasitarias como la malaria, la leishmaniasis y la enfermedad de Chagas tienen una mayor prevalencia. A su vez, en los centros urbanos de las ciudades ubicadas en climas cálidos y templados, prevalecen las arbovirosis reemergentes como el dengue y las emergentes como la del virus del Zika y la fiebre del chikunguña, las cuales son transmitidas por el mosquito *Aedes aegypti*, principal vector transmisor en la región. Estas presentan amplia distribución e intensidad variable de transmisión ^3^. La carga de la morbilidad producida por estas arbovirosis en las áreas urbanas de la región de las Américas en 2022, fue de 3’123.752 casos, y el dengue contribuyó con cerca del 90 % de esta carga ^4^.

En Colombia, se estima que cerca de 40 millones de personas se encuentran en riesgo de adquirir estas arbovirosis ^5^. Desde su reemergencia en el país durante la década de 1970, la endemia del dengue se incrementó alrededor de ocho veces entre 1980 y 2019. Pasó de 108.159 casos en los ochenta, a 876.986 casos en la década del 2010; las formas complicadas representaron alrededor del 5 % y se registró una media anual de 133 muertes en el territorio colombiano entre el 2010 y el 2019 ^6^.

La carga económica y social producida por este evento en el país, se ha estimado en años recientes. Castro-Rodríguez *et al.*, en el 2015, determinaron que, en un año epidémico como el 2010, se perdieron en Colombia 1’198,73 años de vida ajustados por discapacidad (AVAD) por millón de habitantes, frente a 83,88 AVAD en años endémicos. El costo financiero total del dengue en el país, desde una perspectiva social, fue de USD$ 167,8 millones en el 2010, de USD$ 129,9 millones en el 2011 y de USD$ 131,7 millones en el 2012 ^7^.

Uno de los factores determinantes esenciales en la expansión y el mantenimiento del dengue, ha sido la creciente urbanización. Esta ha sido ocasionada, principalmente, por las migraciones poblacionales del campo a la ciudad, huyendo de la violencia por conflictos sociopolíticos o en búsqueda de oportunidades laborales y mejores condiciones de vida. La Comisión Económica para América Latina y el Caribe ha estimado que, actualmente, más del 70 % de la población de los países de las Américas viven en los principales centros urbanos.

Esto ha ocasionado el crecimiento caótico de las zonas marginales periféricas en las ciudades, con una gran proporción de poblaciones vulnerables que viven en precarias condiciones de vida, con insuficiente cobertura y mala calidad de servicios básicos ^8^. La situación genera la necesidad de almacenar agua para uso y consumo humano, problemas de saneamiento ambiental y proliferación de criaderos que mantienen una gran infestación del vector; además, la mayoría son zonas densamente pobladas y en hacinamiento, con desarraigo, baja percepción del riesgo de adquirir la enfermedad, con predominio de conductas y prácticas que favorecen la reproducción de criaderos de *A. aegypti*. La circulación frecuente de diferentes serotipos virales del dengue favorece y mantiene la dinámica e intensidad de la transmisión endemo-epidémica y la aparición de contingencias epidémicas estacionales. La población está expuesta a los serotipos circulantes transmitidos por *A. aegypti*, los cuales favorecen la dinámica de transmisión endemo-epidémica y las recrudescencias frecuentes por estas enfermedades. Además, la globalización ha producido mayor frecuencia y rapidez de los viajes debido a la gran cantidad de intercambios comerciales, desplazamientos poblacionales y turismo, lo que agrava aún más la situación ^9^.

Actualmente, un factor contextual global de enorme relevancia es el cambio climático, el cual contribuye a la exacerbación de la dinámica de la transmisión persistente y la emergencia de eventos nuevos. Se asocia con cambios en la temperatura, las precitaciones y la humedad, que influyen en el ciclo de vida, la tasa de crecimiento, la supervivencia de las larvas y los huevos, y el ciclo reproductivo del mosquito *A. aegypti.* También, afecta la replicación del virus, la maduración y el periodo infeccioso, el desplazamiento de la distribución de las poblaciones de mosquitos y la mayor supervivencia en altitudes más elevadas, la modificación de la frecuencia de picadura, la supervivencia, la reducción o el aumento del periodo de incubación externo, lo cual aumenta la tasa de replicación vírica en el mosquito ^10^.

Asimismo, la malaria o paludismo es otra enfermedad transmitida por vectores prioritaria que, por su magnitud y persistencia histórica, principalmente en los entornos rurales tropicales de las Américas, se ha mantenido como uno de los mayores problemas de salud pública en la mayoría de los países endémicos de la región. Se le considera un evento endemo-epidémico persistente, con una distribución regional heterogénea y focalizada, inestable y de escasa transmisión. Ocurre en espacios receptivos donde la interacción socioecológica de las poblaciones expuestas y las portadoras de parásitos, y la presencia de vectores ocasionada por alteraciones en la receptividad y vulnerabilidad, favorecen las dinámicas de transmisión. Esta prevalece en zonas rurales receptivas de 17 países de la región. Durante los años transcurridos en el presente milenio, hubo una reducción de la carga de malaria de unos 900.000 casos, pasando de 1,5 millones en el 2000, a 600.000 reportados en el 2021. En los países endémicos de la región, se presenta un espectro de situaciones epidemiológicas que incluye los pocos que la han eliminado o están en proceso de hacerlo, algunos que registran resurgencia de la transmisión y otros que no muestran cambios aparentes ^11^.

En Colombia, entre 1978 y 2021, se registró un acumulado de 4’505.552 casos de malaria, correspondientes a una media anual de 102.399 casos. El comportamiento general de la morbilidad por malaria en Colombia mantuvo un patrón endemo-epidémico variable en el período 1978-2021, observándose una acentuada tendencia al ascenso entre 1978 y 1999, seguida de un progresivo descenso a partir de la primera década del presente siglo hasta el 2021. La reducción de nuevos casos se produjo a expensas del componente endémico, pero no hubo cambios en la frecuencia o en la intensidad de la transmisión epidémica. En el mismo periodo de 1978 a 2021, la mortalidad por malaria mantuvo un descenso importante y sostenido en el país (Padilla- Rodríguez JC, Olivera M, Acuña L, Chaparro P. Cambios en la endemo-epidemia de la malaria en Colombia, 1978-2021. En prensa).

Recientemente, se ha estimado la carga económica institucional y social producida por la malaria en el país. Olivera *et al*. calcularon que, en el 2019, considerado un año epidémico, se registraron 80.415 casos de malaria y los costos totales atribuibles a esta enfermedad fueron de USD$ 2,1 millones, con diferencias sustanciales entre los regímenes de seguro (USD$ 1,9 millones para el régimen subsidiado y USD$ 191.973,4 para el régimen contributivo). El costo promedio de atención de la malaria no complicada en el país fue de USD$ 22,5. Los costos de atención de los casos no complicados por especies parasitarias fue de USD$ 17,5 para las infecciones por *P. falciparum*, USD $ 17,4 para *P. vivax* y de USD$ 17,3 para las infecciones mixtas. En cambio, el costo de la atención de la malaria complicada para todas las edades y especies fue, en promedio, de USD$ 593,75 ^12^.

La transmisión endemo-epidémica y persistente de la malaria ha evolucionado históricamente en forma variable en los diferentes espacios o territorios receptivos y endémicos de transmisión en el territorio nacional, y ha estado ligada con el desarrollo social y económico del país. Actualmente, entre los principales factores determinantes que explican la dinámica de dicha transmisión, se encuentran: la explotación ilegal, intensiva e irracional de los recursos naturales y minerales, en regiones receptivas; la expansión y concentración de áreas de cultivos de uso ilícito en regiones endémicas, con abundante biomasa vegetal y gran riqueza hídrica, la cual hace parte actualmente del continuo e inacabado proceso de ampliación de la frontera agrícola. Esto ha conllevado deforestación permanente de las selvas y deterioro del medio ambiente, generando riesgos que favorecen la proliferación de criaderos y el incremento de la densidad de la población de anofelinos competentes que mantienen la endemia local y la aparición de brotes estacionales frecuentes. A su vez, la intensa migración interna de grupos poblacionales no inmunes procedentes de áreas no endémicas hacia regiones rurales endémicas -donde existen centros de explotación de cultivos ilícitos y de minería ilegal con condiciones adecuadas para la existencia de una abundante fauna anofelínica, cuyas interacciones socio- ecológicas- favorecen la aparición de epidemias estacionales y explosivas frecuentes. Además, desde estos focos de transmisión activa, se producen flujos de dispersión poblacional de portadores (sintomáticos o asintomáticos) de parásitos hacia otras regiones receptivas del territorio nacional, lo cual contribuye al resurgimiento de focos de transmisión ^13^.

En Colombia, desde la primera mitad del siglo XX, se han importado y adoptado iniciativas políticas de control y erradicación de enfermedades como la uncinariasis, la fiebre amarilla urbana, el dengue, la malaria, el pian y el sarampión, entre otras, promovidas desde sus inicios por instituciones internacionales de cooperación técnica, lográndose algunos resultados exitosos puntuales y otros fugaces e insostenibles. Un ejemplo de esto fueron las campañas de erradicación del dengue y la malaria, en las décadas de los cincuenta y sesenta, respectivamente, las cuales mostraron resultados positivos inicialmente, pero que no fueron sostenibles en el tiempo ^14^. El estruendoso fracaso se atribuyó, principalmente, a problemas técnicos y administrativos durante la gestión de las campañas. A pesar de esto, este paradigma hegemónico ha sido revitalizado y readecuado para insistir en la eliminación de la malaria, casi que con la misma estrategia, en el mediano plazo.

Este modelo hegemónico, biologicista, reduccionista, prescriptivo, monológico y tecnocrático, ha demostrado ser insuficiente para intentar un abordaje más holístico e integral que responda a la complejidad y multidimensionalidad de estos problemas. Además, predominan la percepción y la convicción de que este tipo de problemas solo se abordan con soluciones tecnológicas de tipo bala mágica y, en realidad, se le presta poca atención e importancia a la voluntad y el compromiso político permanente de los actores sociales e institucionales responsables del problema y la solución. Pese a esto, los organismos de cooperación internacional que lideran las políticas mundiales y regionales, mantienen el enfoque y lo remozan coyunturalmente, y siguen recomendando a los países endémicos su adopción. Estos lo adoptan y aplican, la mayoría de las veces de forma acrítica y empírica, perpetuando una especie de círculo vicioso y la sensación de un *deja vú*.

Finalmente, teniendo en cuenta que las enfermedades transmitidas por vectores son problemas complejos, multidimensionales, dinámicos y prioritarios de salud pública, el reto más inmediato que enfrentamos sería proponer y desarrollar modelos innovativos de intervención, que aborden la complejidad de estos eventos de forma integral, multidisciplinaria y transdisciplinaria, bajo el liderazgo del sector salud, garantizando la participación de los actores institucionales y sociales responsables en forma dialógica, así como optimizando el uso inteligente de la tecnología costo- efectiva disponible y el desarrollo institucional, la capacitación y la generación de masa crítica y el desarrollo de la investigación básica y aplicada. Además, se requiere optimizar los recursos existentes y la búsqueda de fuentes adicionales de financiación, para la mejora continua de la capacidad institucional que conlleve el fortalecimiento de la respuesta técnica operativa permanente de las acciones de prevención y el control oportuno.
